# Measuring microRNA reporter activity in skeletal muscle using hydrodynamic limb vein injection of plasmid DNA combined with *in vivo* imaging

**DOI:** 10.1186/2044-5040-3-19

**Published:** 2013-08-01

**Authors:** Martin G Guess, Kristen KB Barthel, Emily K Pugach, Leslie A Leinwand

**Affiliations:** 1 Department of Molecular, Cellular and Developmental Biology and BioFrontiers Institute, University of Colorado, Boulder, CO, USA

**Keywords:** MicroRNA, *In vivo* imaging, Hydrodynamic limb vein injection, Plasmid DNA, Reporter assay, Gene therapy, Muscular dystrophy, Luciferase, Bioluminescence

## Abstract

**Background:**

microRNA regulation plays an important role in the remodeling that occurs in response to pathologic and physiologic stimuli in skeletal muscle. In response to stress, microRNAs are dynamically regulated, resulting in a widespread “fine-tuning” of gene expression. An understanding of this dynamic regulation is critical to targeting future therapeutic strategies. Experiments elucidating this dynamic regulation have typically relied on *in vitro* reporter assays, *ex vivo* sample analysis, and transgenic mouse studies. Surprisingly, no experimental method to date allows rapid *in vivo* analysis of microRNA activity in mammals.

**Methods:**

To improve microRNA studies we have developed a novel reporter assay for the measurement of skeletal muscle microRNA activity *in vivo*. To minimize muscle damage, hydrodynamic limb vein injection was used for the introduction of plasmid DNA encoding bioluminescent and fluorescent reporters, including click-beetle luciferase and the far-red fluorescent protein mKATE. We then applied this technique to the measurement of miR-206 activity in dystrophic *mdx4cv* animals.

**Results:**

We found that hydrodynamic limb vein injection is minimally damaging to myofibers, and as a result no induction of muscle-specific miR-206 (indicative of an injury response) was detected. Unlike intramuscular injection or electroporation, we found that hydrodynamic limb vein injection results in dispersed reporter expression across multiple hindlimb muscle groups. Additionally, by utilizing click-beetle luciferase from *Pyrophorus plagiophthalamus* as a reporter and the far-red fluorescent protein mKATE for normalization, we show as a proof of principle that we can detect elevated miR-206 activity in *mdx4cv* animals when compared to C57Bl/6 controls.

**Conclusion:**

Hydrodynamic limb vein injection of plasmid DNA followed by *in vivo* bioluminescent imaging is a novel assay for the detection of reporter activity in skeletal muscle *in vivo.* We believe that this method will allow for the rapid and precise detection of both transcriptional and post-transcriptional regulation of gene expression in response to skeletal muscle stress. Additionally, given the post-mitotic status of myofibers and stable expression of plasmid DNA, we believe this method will reduce biological variability in animal studies by allowing longitudinal studies of the same animal cohort.

## Background

Skeletal muscle displays a remarkable ability to remodel in response to pathologic and physiologic stimuli ([1-3] and reviewed in [4]). How changes in gene expression mediate these processes by post-transcriptional regulation has been heavily studied in the context of diseased, hypertrophic, and regenerating skeletal muscle [5-7]. While recent studies have greatly enhanced our understanding of these dynamic processes, measuring post-transcriptional changes that occur *in vivo* is challenging and this has limited the experimental avenues available. Additionally, the discovery of post-transcriptional regulation by microRNAs (miRNAs) in skeletal muscle has increased the complexity of these processes.

miRNAs are a type of short, non-coding RNA that post-transcriptionally regulate gene expression. miRNAs function by guiding the RNA-induced silencing complex (RISC) to target mRNAs, dictated by base pairing to target sites primarily in their 3′-untranslated regions (UTRs). miRNAs can downregulate the expression of several to hundreds of target genes by repressing translation and/or destabilizing target mRNAs [8,9]. miRNAs are required for normal skeletal muscle development in mice [10], and several miRNAs have been shown to be dynamically regulated during hypertrophy [11], acute exercise [12], regeneration after injury [13], and in the remodeling that occurs in response to genetic muscle disease [14-17]. In particular, miR-206 is expressed specifically in skeletal muscle, and is highly expressed in regenerating fibers [13]. miR-206 has been shown to promote terminal differentiation of myoblasts by regulating the expression of genes including connexin43 [18], utrophin [19], pax3 [20], pax7 [21] and DNA polymerase α [22]. Many of these studies rely on data obtained from transgenic mouse studies, and *ex vivo* sample analysis - both currently invaluable to the study of miRNAs. The study of miRNA regulation in skeletal muscle, however, would benefit from a system that enabled rapid, reproducible, longitudinal *in vivo* reporter assays, at a fraction of the cost of transgenic mouse production and analysis, and with fewer animals needed than for *ex vivo* studies.

Several studies have shown that stable gene expression can be achieved in post-mitotic myofibers *in vivo* by the introduction of plasmid DNA (pDNA) [23-26]. Unlike viral DNA vectors, pDNA is non-immunogenic [23], easily manipulated, and relatively inexpensive to produce. Methods for introducing naked pDNA into myofibers include intramuscular injection [24], electroporation [26,27], and more recently, hydrodynamic limb vein (HLV) injection [28,29]. While these methods all result in efficient myofiber transduction, only HLV injection is minimally damaging to the muscle [30], enabling studies of muscle remodeling to take place in the absence of widespread myofiber regeneration. Moreover, HLV injection results in more widespread pDNA distribution than intramuscular injection or electroporation, which are limited to the site of injection, or single muscle groups, respectively [24,26].

*In vivo* bioluminescent and fluorescent imaging (BLI) of genetically encoded reporters has been a useful tool in murine xenograft studies including cancer [31], chondrogenic differentiation [32], viral infection [33], and even in studies of miRNA biogenesis and post-transcriptional regulation [34,35]. All of these studies, however, have relied on the expression of reporter genes in cells cultured *in vitro*, and none have demonstrated the ability to quantify skeletal muscle reporter gene expression *in situ.* We therefore developed a novel system using HLV injection of reporter pDNA into skeletal muscle in combination with *in vivo* BLI in order to quantify miRNA activity. Before testing, we anticipated that animal-to-animal variation in injection efficiency would confound true changes in reporter expression. While several groups have reported the utility of *Renilla* luciferases as a normalizer in *in vivo* reporter studies [32,36], we found that the fast kinetics and requirement for intravenous substrate delivery made this approach technically challenging. To solve this problem, we employed a dual-reporter approach consisting of a high-efficiency click-beetle luciferase from *Pyrophorus plagiophthalamus* (CBG99) [37], and the far-red fluorescent protein mKATE [38] for signal normalization. mKATE has been shown to have a maximum emission wavelength (635 nm) that is optimal for tissue penetration, and to have high pH and photostability, making this an ideal protein for *in vivo* BLI.

As a proof of principle, we measured miR-206 activity and show that the activity measured using this technique reproduces the levels obtained from quantitative reverse transcription PCR (qRT-PCR) measurements. We believe this technique will prove useful not only in the quantification of real-time miRNA activity, but also in studies of transcriptional and post-transcriptional regulation.

## Methods

### Plasmid construction

Two perfectly complementary miR-206 [NCBI:NR_029593] binding sites were inserted between the Xba1 and Fse1 restriction sites downstream of the CBG99 stop codon in pCBG99-Control (Promega, Madison, WI, USA) using the following oligonucleotide sequences: 5′-CTAGACCACACACTTCCTTACATTCCAAAACCACACACTTCCTTACATTCCAGGCCGG, and 5′-CCTGGAATGTAAGGAAGTGTGTGGTTTTGGAATGTAAGGAAGT GTGTGGT. pcDNA-mKATE was a kind gift from Amy Palmer (University of Colorado at Boulder, Boulder, CO, USA), and pCMV-eGFP from Stephen Langer (University of Colorado at Boulder, Boulder, CO, USA).

### Hydrodynamic limb vein injection

All animal experiments were performed using protocols approved by University of Colorado and Colorado State University Institutional Animal Care and Use Committees (IACUC). Mice were injected according to a protocol modified from Hagstrom, *et al*. [29]. Briefly, after sedation with 1 to 4% inhaled isoflurane, a tourniquet was secured around the upper hindlimb to restrict blood flow for 1 to 2 minutes prior to injection, and 2 minutes after injection. The hindlimb was first cleaned with 70% ethanol, and an incision was made with surgical scissors on the medial surface of the leg to expose the great saphenous vein. A ½ inch 30-gauge needle connected by catheter to a syringe was then inserted in an anterograde direction into the vein. Endotoxin-free pDNA isolated using Endofree® Plasmid Maxi Kit (Qiagen, Hilden, Germany) and diluted in sterile saline solution (volume determined according to the formula: 1 + (((body weight in grams − 25)/25) × 1/2)mL), was delivered at a rate of 7 mL/minute by a programmable syringe pump (KD Scientific, Holliston, MA, USA). Two minutes after the injection was completed, the tourniquet was released and the incision was closed with nonabsorbable 6–0 silk suture (Davis-Geck, Brooklyn, NY, USA). Mice recovered on a 37°C heat block and were monitored for adverse effects.

### Barium chloride injury

To induce muscle degeneration, mice were first anesthetized with 1 to 4% inhaled isoflurane, and the right hindlimb was shaved. After cleaning the area with 70% ethanol, the right gastrocnemius was injected with 50 μL of a 1.2% barium chloride solution in normal saline using a 27-gauge insulin syringe. Mice recovered on a 37°C heat block and were monitored for adverse effects.

### *In vivo* bioluminescent and fluorescent imaging

Animals were anesthetized using 1 to 4% isoflurane prior to imaging and placed in the chamber of an IVIS 100 *in vivo* imaging system (Caliper Biosciences, Hopkinton, MA, USA) housed at Colorado State University’s Animal Cancer Center (Fort Collins, CO, USA). The mKATE fluorescent signal was then collected using sequential mode with a 1-s exposure time and the Cy5.5 excitation/emission filter set. Animals were then removed from the imaging chamber and injected intraperitoneally with 200 μL of 30 mg/mL D-luciferin. Ten minutes after substrate injection, bioluminescent signal was collected for 1 minute using the open filter mode. Images were analyzed using LivingImage software (Caliper Biosciences) and photons/sec/cm^2^/steradian were quantified using region-of-interest (ROI) analysis. Mice recovered from anesthesia on a 37°C heat block.

### Immunofluorescence

Muscles for immunofluorescence were frozen in liquid nitrogen-cooled isopentane and mounted in optimal cutting temperature (OCT) medium (Sakura Finetek, Torrance, CA, USA). Cryosections (12 μm thick) were fixed in 4% paraformaldehyde for 10 minutes at room temperature, then blocked for 1 hour with 5% goat serum in PBS and 0.1% Triton X-100. Following blocking, sections were stained with anti-laminin at 1:500 (L-9393, Sigma, St. Louis, MO, USA) or anti-desmin at 1:20 (D-8281, Sigma) overnight at 4°C. After washing several times in PBS with 0.1% Triton X-100, anti-rabbit Texas red secondary antibody was applied at 1:100 dilution for 1 hour at 37°C (711-075-152, Jackson, West Grove, PA, USA). After washing again in PBS with 0.1% Triton X-100, sections were counterstained with 300 nM 4′,6-diamidino-2-phenylindole (DAPI) for 5 minutes at room temperature (Sigma D-9542), and mounted using Fluoromount G (Southern Biotech, Birmingham, AL, USA).

### Quantification of injection efficiency

To determine HLV injection efficiency, percentages of GFP-positive fibers were quantified using immunofluorescence on lower hindlimb sections from pCMV-eGFP-injected animals. Following imaging on an inverted epifluorescent microscope (Eclipse TE2000, Nikon, Melville, NY, USA), GFP, laminin, and DAPI images were merged to produce a composite (ImageJ software, National Institutes of Health, Bethesda, MD, USA). GFP-positive fibers were counted when GFP signal across a myofiber was greater than background levels. Total fiber number was counted using laminin staining to demarcate fiber boundaries. Percentages are reported as an average of GFP-positive fibers for ten fields of view for the gastrocnemius, and five fields each for the soleus and tibialis anterior (TA).

### qRT-PCR

To measure miRNA expression, total RNA was first isolated from snap-frozen skeletal muscles using TRI reagent (Molecular Research Center, Cincinnati, OH, USA). Then, 7 ng of total RNA per reaction was reverse-transcribed and PCR-amplified on a CFX96 thermocycler (Bio-Rad, Hercules, CA, USA) using Taqman miRNA assays (Invitrogen, Grand Island, NY, USA). Relative miRNA expression was determined using the 2^-ΔΔCt^ method [39], using sno202 as a reference gene.

### Statistical analysis

For qRT-PCR experiments, relative miR-206 expression values were compared to controls (for assessment of muscle damage, controls were uninjured/uninjected contralateral limbs for each time point, and for miR-206 reporter measurements, controls were C57Bl/6 animals) using the unpaired Student’s *t-*test. For BLI measurements, statistics were performed by comparing mean normalized photons/s/cm^2^/steradian (CBG99/mKATE) of pCBG99-2x-miR-206-injected hindlimbs, to mean normalized photons/s/cm^2^/steradian of pCBG99-Control-injected hindlimbs for *mdx4cv* and C57Bl/6 animals using the unpaired Student’s *t*-test.

## Results

### HLV injection does not induce myofiber regeneration

**Figure 1:**
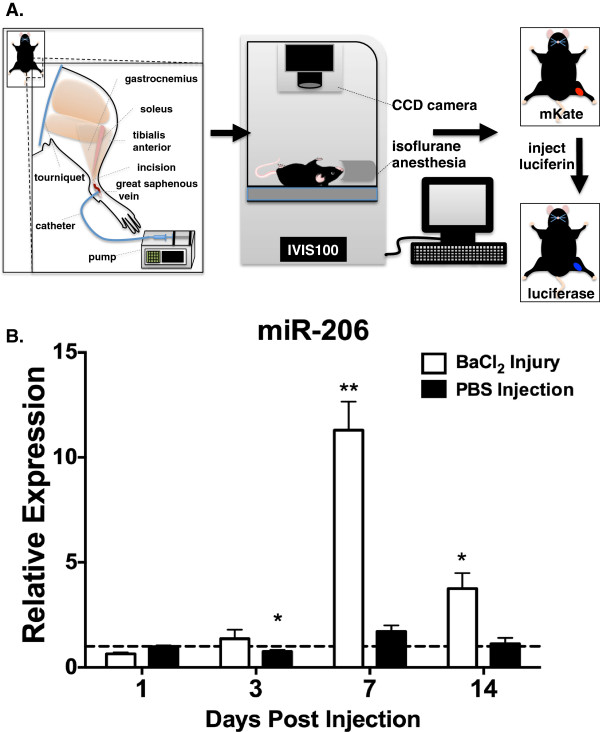
**miR-206 levels after hydrodynamic limb vein injection. (A)** Schematic depiction of hydrodynamic limb vein injection (HLV) of plasmid DNA followed by *in vivo* bioluminescent and fluorescent imaging (BLI). **(B)** miR-206 expression in 3 to 4 month-old C57Bl/6 mice receiving either HLV injection of saline solution or BaCl_2_ injury, sacrificed at the indicated time points, normalized to sno202. Relative levels measured in the right (treated) gastrocnemius are displayed as mean values normalized to contralateral controls; n= 3 or 4 animals/group, Error bars = standard error of the mean. ^*^P ≤0.05, ^**^P ≤0.001.

Prior to employing HLV to deliver miRNA reporters for miR-206 activity, it was important to determine whether the injection technique itself would induce muscle regeneration and consequently increase the levels of miR-206. To test this, we performed HLV injection (Figure 1A) on wild-type C57Bl/6 mice using saline only and compared miR-206 expression to that in animals injured with BaCl_2_. miR-206 levels decreased slightly (*P* =0.02) on the third day after saline injection, likely due to residual edema from the high-volume injection, but remained unchanged (day 7 animals trended towards a 1.7-fold increase, *P* = 0.051) on subsequent days (Figure 1B). Conversely, barium chloride injury strongly induced miR-206 approximately 11-fold (*P* = 0.0003) as previously reported [13], suggesting that HLV injection does not activate a widespread program of muscle regeneration.

### Reporter expression is well distributed and is quantifiable using *in vivo* imaging

**Figure 2:**
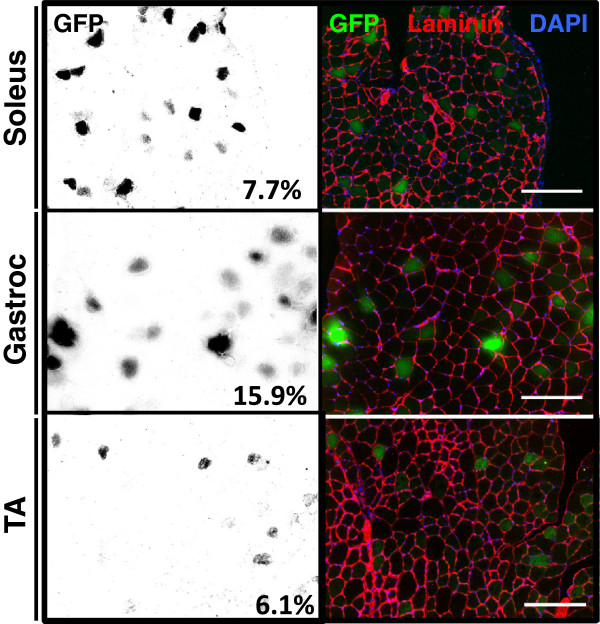
**Reporter distribution.** Reporter expression after hydrodynamic limb vein injection. Left column: green fluorescent protein (GFP) epifluorescent signal displayed as inverted grayscale image, percentages are an average of GFP-positive fibers for indicated muscles. Right column: immunostaining for laminin (red) and epifluorescence for GFP (green) and 4′,6-diamidino-2-phenylindole (DAPI) (blue) to show distribution of GFP-positive myofibers in indicated muscles; n=1 animal. Scale bars = 100 μm. TA, tibialis anterior.

**Figure 3:**
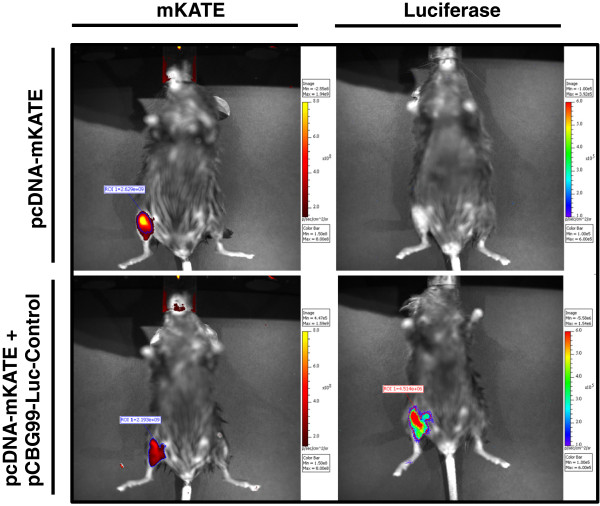
**Reporter detection using *in vivo *bioluminescent imaging.** Mice were injected with pcDNA-mKATE (top) or pcDNA-mKATE and pCBG99-Luc-Control (bottom). Data collected using Cy5.5 excitation/emission filters is shown in the left column, and after D-luciferin injection in the right column.

To test the muscle distribution of reporter pDNA, we next injected C57Bl/6 mice with 100 μg/animal of pCMV-eGFP. After 7 days, we performed immunofluorescence on fixed cryosections and found that GFP expression was visible in the sarcoplasm of myofibers in the gastrocnemius, soleus, and TA muscles (Figure 2). To determine injection efficiency, we quantified GFP-positive myofibers and found that 7.7%, 15.9% and 6.1% of fibers in the soleus, gastrocnemius, and TA, respectively, expressed the reporter. We also observed an absence of centrally located nuclei in GFP-positive myofibers, further supporting the finding that HLV injection itself causes minimal muscle injury. Next, we tested whether reporter gene expression can be quantified from both bioluminescent and fluorescent reporters using *in vivo* imaging. To this end, we injected either 100 μg each of pCBG99-Luc-Control and pcDNA-mKATE, or pcDNA-mKATE alone into the right hindlimbs of C57Bl/6 mice and collected images 7 days later using BLI. The signal was easily visible and localized to the hindlimb skeletal muscle for both reporters, although mKATE background signal was frequently observed from the ventilation nosepiece and/or the distal parts of the hindlimb (Figure 3). We also found that mKATE fluorescence did not bleed into the luciferase channel, making it ideal for *in vivo* use in combination with CBG99-luciferase.

### miRNA activity measurements are consistent with miRNA qRT-PCR measurements

**Figure 4:**
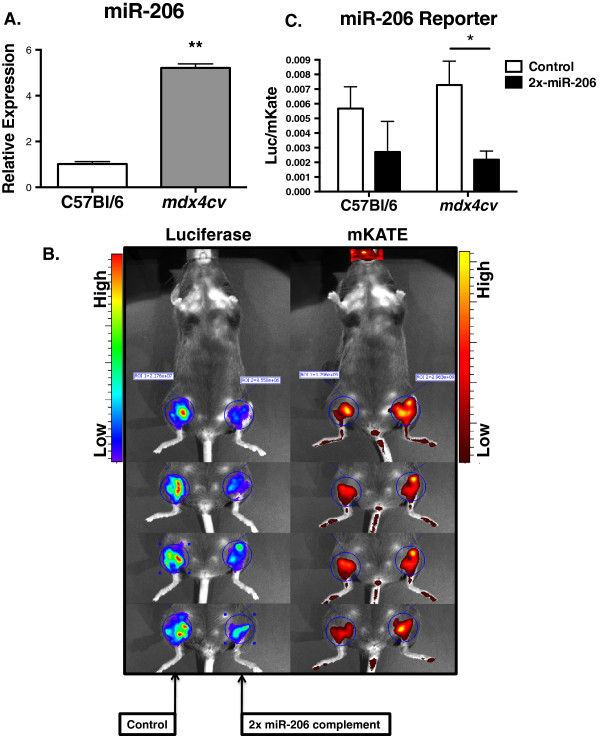
**Quantification of miR-206 reporter activity in *mdx4cv *mice. (A)** miR-206 expression measured in the gastrocnemius muscles of 3 month-old *mdx4cv* animals in comparison to C57Bl/6. miR-206 expression is normalized to sno202 ( n = 4 animals/group), mean values are displayed; error bars *=* standard error of the mean (SEM), ^*^P ≤0.05. **(B)** Bioluminescent imaging of *mdx4cv* animals showing CBG99 luciferase and mKATE expression. Left hindlimbs were co-injected with pCBG99-2x-miR-206 and pcDNA-mKATE, and right hindlimbs were co-injected with pCBG99-Control and pcDNA-mKATE. **(C)** Region of interest (ROI) analysis of **(B)**. The same region size was used for all animals (n= 4 animals/group; mean values are displayed; error bars = SEM, *P≤0.05.

**Figure 5:**
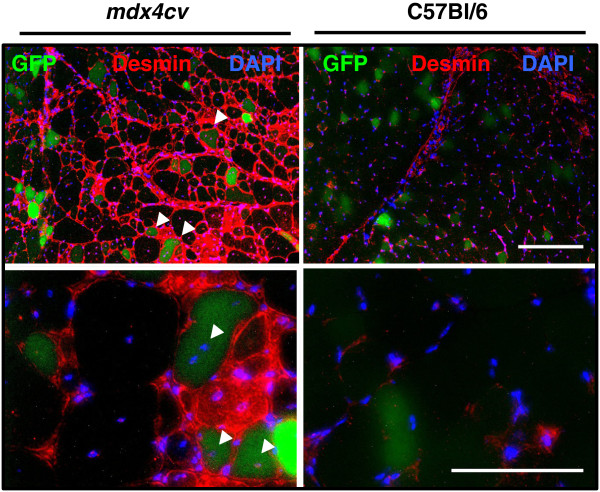
**Reporter expression in regenerating *mdx4cv *fibers.** pCMV-eGFP-injected *mdx4cv* (left column) and C57Bl/6 (right column) gastrocnemius sections showing desmin immunostaining (red), green fluorescent protein (GFP) epifluorescence (green), and 4′,6-diamidino-2-phenylindole (DAPI) (blue) demonstrate GFP expression in regenerating fibers. Upper row images were taken using 10× objective lens, lower row with 20× objective. Scale bars = 100 μm.

In order to measure miRNA activity *in vivo,* we inserted two perfectly complementary miRNA binding sites downstream of the CBG99 luciferase coding sequence, creating pCBG99-2x-miR-206. We next co-injected 100 μg of pcDNA-mKATE along with either pCBG99-2x-miR-206 (left hindlimbs) or pCBG99-Luc-Control (right hindlimbs) into C57Bl/6 or dystrophic *mdx4cv* mice. Seven days later, we measured the signal using *in vivo* BLI followed by ROI analysis (Figure 4B). To ensure that we minimized the effect of differential plasmid distribution and injection efficiency, the same region size was used for quantification in all animals, and the luciferase signal was normalized to mKATE. After normalizing, the CBG99:mKATE ratio from left hindlimbs was compared to control right hindlimbs. In agreement with a 5-fold increase in miR-206 expression in *mdx4cv* animals by qRT-PCR (*P* = 0.0001) (Figure 4A), the normalized bioluminescent signal in the left hindlimbs of *mdx4cv* mice was reduced 3.3-fold (*P* = 0.02) (Figure 2C). While the decreased average signal in C57Bl/6 did not reach significance *(P =* 0.15), the downward trend is likely due to the high abundance of miR-206 in skeletal muscle, and the decreased miR-206 reporter signal in *mdx4cv* animals is due to increased miR-206 expression. Given that miR-206 is highly expressed in the regenerating fibers of dystrophic mice [13], this result suggests that pDNA injected using HLV is also expressed in these fibers. To test this, we injected *mdx4cv* animals with 100 μg of pCMV-eGFP and collected tissues for immunofluorescence 7 days later. As expected, we found GFP expression in small, desmin-positive, regenerating myofibers with centrally located nuclei (Figure 5, arrowheads), indicating that the decrease in luciferase signal measured using BLI likely corresponds to a loss of luciferase activity in these fibers.

## Discussion

Several groups have reported the safety and efficacy of pDNA injections using HLV for gene therapy approaches [23,28,29]. We found this technique also to be useful in studies requiring minimally damaging introduction of genetically encoded reporters into skeletal muscle and have used it in combination with BLI and ROI analysis to analyze *Myh7b* promoter activity *in vivo*[40]. We also found that this technique induces minimal muscle regeneration as assessed by miR-206 induction, and to our knowledge this is the first report of using an *in vivo* transfection method that does not induce myofiber regeneration for luciferase reporter studies.

The distribution of GFP-positive myofibers after HLV injection that we have seen is similar to that reported by Wooddell, *et al*. [41] although the percentage of transfected fibers we report is lower. This may be due to decreased sensitivity of detecting a fluorescent reporter (GFP) versus a colorimetric stain (β-galactosidase). Similar to this report, we find the highest percentage (15.9%) of transfected myofibers in the posterior lower leg (gastrocnemius) muscles and the lowest percentage (6.1%) in the anterior lower leg (TA) muscles (Figure 3). Additionally, others have observed reporter gene expression up to 49 weeks after HLV injection [41], suggesting that this method could be used for longer-term regulatory studies, provided that the luciferase and mKATE signals follow similar expression profiles.

## Conclusion

In summary, HLV injection of pDNA reporters into skeletal muscle followed by BLI is a useful technique for studies of gene regulation. Here, we show that it is possible to measure miRNA activity in myofibers *in situ,* without activation of a regeneration response. This technique has the added benefit of reducing the cost associated with producing transgenic animals for skeletal muscle studies, and will likely allow reduced animal numbers and decreased variability in future experiments by enabling longitudinal studies of the same animal cohort.

## Abbreviations

BLI: Bioluminescent and fluorescent imaging; DAPI: 4′,6-diamidino-2-phenylindole; GFP: Green fluorescent protein HLV: hydrodynamic limb vein; miRNA: microRNA; OCT: Optimum cutting temperature; PBS: Phosphate-buffered saline; pDNA: Plasmid DNA; qRT-PCR: Quantitative reverse transcription polymerase chain reaction; RISC: RNA-induced silencing complex; ROI: Region of interest; UTR: Untranslated region; TA: Tibialis anterior.

## Competing interests

The authors have no competing interests to declare.

## Authors’ contributions

MG designed the experiments, wrote the paper, and carried out the qRT-PCR, injections, imaging, and molecular cloning. KB played a key role in the project’s conception and provided useful discussion and critical reading of the manuscript. EP provided assistance with *in vivo* BLI and assembling figures. LL provided mentoring and discussion about all experiments and data. All authors have read and approved the manuscript.
